# Impact of Outdoor Air Pollution on Indoor Air Quality in Low-Income Homes during Wildfire Seasons

**DOI:** 10.3390/ijerph16193535

**Published:** 2019-09-21

**Authors:** Prateek M. Shrestha, Jamie L. Humphrey, Elizabeth J. Carlton, John L. Adgate, Kelsey E. Barton, Elisabeth D. Root, Shelly L. Miller

**Affiliations:** 1 Department of Mechanical Engineering, University of Colorado Boulder, Boulder, CO 80309, USA; prateek.shrestha@colorado.edu (P.M.S.); jlh563@drexel.edu (J.L.H.); 2 Department of Environmental and Occupational Health, University of Colorado, Colorado School of Public Health, Aurora, CO 80045, USA; ELIZABETH.CARLTON@ucdenver.edu (E.J.C.); john.adgate@ucdenver.edu (J.L.A.); KELSEY.BARTON@ucdenver.edu (K.E.B.); 3 Department of Geography and Division of Epidemiology, The Ohio State University, 1036 Derby Hall, 154 North Oval Mall, Columbus, OH 43210, USA; root.145@osu.edu

**Keywords:** low-cost sensors, black carbon, PM_2.5_, infiltration, energy efficiency, traffic-related air pollution, wildfire smoke

## Abstract

Indoor and outdoor number concentrations of fine particulate matter (PM_2.5_), black carbon (BC), carbon monoxide (CO), and nitrogen dioxide (NO_2_) were monitored continuously for two to seven days in 28 low-income homes in Denver, Colorado, during the 2016 and 2017 wildfire seasons. In the absence of indoor sources, all outdoor pollutant concentrations were higher than indoors except for CO. Results showed that long-range wildfire plumes elevated median indoor PM_2.5_ concentrations by up to 4.6 times higher than outdoors. BC, CO, and NO_2_ mass concentrations were higher indoors in homes closer to roadways compared to those further away. Four of the homes with mechanical ventilation systems had 18% higher indoor/outdoor (I/O) ratios of PM_2.5_ and 4% higher I/O ratios of BC compared to other homes. Homes with exhaust stove hoods had PM_2.5_ I/O ratios 49% less than the homes with recirculating hoods and 55% less than the homes with no stove hoods installed. Homes with windows open for more than 12 hours a day during sampling had indoor BC 2.4 times higher than homes with windows closed. This study provides evidence that long-range wildfire plumes, road proximity, and occupant behavior have a combined effect on indoor air quality in low-income homes.

## 1. Introduction

Homes are meant to keep us safe against undesirable natural elements including outdoor air pollution. We spend the majority of our time indoors and often at home, and thus exposure to air pollution in our homes is an ongoing concern [1]. Indoor air quality is degraded by air pollutants of both indoor and outdoor origins. In addition to indoor emissions from activities like cooking and cleaning, a major source of air pollution in homes is the infiltration of outdoor air pollutants. Infiltration refers to the transport of air due to pressure and temperature differences in and out of homes through unintentional openings. Outdoor air pollution can also come indoors through open windows and doors or by supply air ventilation systems. Every year, as climate change continues to impact our environment in addition to rapid population growth and increased anthropogenic emissions, outdoor air quality impacts include increases in summertime ozone in urban areas, particulate matter due to more frequent wildfires, and airborne pollens, molds and biogenic volatile organic compounds [2,3,4,5], making exposure to outdoor air pollution more and more extreme and resulting in higher health risks, especially in the sensitive receptors of urban areas. Indoor environments of homes are the most obvious first line of defense against these air pollutants. In this study, we continuously monitored indoor and outdoor air pollutants in 28 low-income homes to study the impacts of outdoor air pollutants on indoor air quality during wildfire seasons.

The highest levels of outdoor air pollution in the Denver metro area of Colorado can be expected during the summer season (the months of June through October of every year) [6]. During this time of the year, outdoor particulate matter levels are elevated due to the usual background level of traffic-related emissions superimposed with atmospheric chemistry processes [7,8,9] and aerosols produced by both short- and long-range wildfires, which are increasing in number over the decades as a result of climate change [10,11]. Studies have shown that wildfire smoke can be transported by wind and can affect the air quality, visibility, and atmospheric chemistry of places that are hundreds of kilometers away from the locations of wildfires [12,13,14,15]. The plumes from wildfires that occur annually during this time in the western United States and Canada can significantly affect the Denver metro area of Colorado.

Infiltration rates of outdoor fine particulate matter in the size range of 2.5 microns (PM_2.5_), like those emitted from wildfires, are known to be higher in homes compared to the coarse and ultrafine particulate matter size ranges [16,17,18]. Wildfire smoke-related PM_2.5_ can take a few minutes to a few hours to infiltrate indoors but can persist for up to eight to ten hours [19]. Outdoor PM_2.5_ can infiltrate indoors in buildings even with closed windows and merely staying indoors provides limited protection against outdoor PM [20]. Many past studies have concluded that wildfire PM_2.5_ are important sources of adverse respiratory health outcomes [21,22,23] and staying indoors combined with the use of air cleaners can effectively reduce PM_2.5_ exposure during wildfire seasons [19,24,25,26]. In many communities, the use of air cleaning technology is often overlooked due to cost and a lack of information.

In addition to the worsening of outdoor air by wildfire plumes, people living in urban homes situated near a major road or a highway are exposed to significantly higher levels of traffic-related air pollutants like black carbon (BC), carbon monoxide (CO), and nitrogen dioxide (NO_2_)—all of which have been associated with adverse health effects including the increased risk of cardiovascular diseases, stroke, and reduced life expectancy [27,28,29]. NO_2_ is a known respiratory tract irritant and marker for traffic-related air pollution [30]. Results from our Colorado Home Energy Efficiency and Respiratory Health (CHEER) study showed that residents living in low-income single-family homes near major roads report more adverse respiratory symptoms compared to residents who live farther than 200 m away [31].

People living in the same geographic location are similarly affected by outdoor air pollution. However, low-income populations are often more vulnerable to the effects of climate change and outdoor air pollution due to financial constraints which compromise their ability to mitigate or adapt to changing environmental conditions which impact health [32,33,34]. Many low-income communities are also disproportionally located in areas with poor environmental conditions and close to high-traffic roads [35,36,37], leading to the unequal burden of potential health impacts. Low-income communities are therefore an important, and underrepresented, community to consider. However, there are limited data related to the indoor air quality in low-income homes, which this study aimed to fulfill.

The key objectives of this study were to better understand how the indoor air quality of low-income homes is impacted by (1) outdoor air pollutants during wildfire seasons when they can be expected to be at their maximum levels, and (2) what role certain characteristics of homes and occupant behavior play to worsen or mitigate those impacts.

## 2. Materials and Methods

This section describes the various materials and methods used in the study.

### 2.1. Study Recruitment

Households located in Denver and the northern front range of Colorado were recruited for this part of the CHEER study through letters mailed to homes that met the low-income criteria set by Low-Income Energy Assistance Program (LEAP) in the state of Colorado. This mailing was accomplished through a partnership with Xcel Energy Inc. and Boulder Housing Partners [38]. Homes were recruited for the study only if all the home occupants were nonsmoking to eliminate smoking as a source of bias in the collected dataset.

Once a home was recruited, a home visit was conducted between the months of June through October. We focused our visits as much as possible on days when outdoor air pollution was elevated due to short- and long-range smoke from wildfires. Home visits lasted approximately two hours during which blower door tests were performed to assess home air tightness, air quality monitoring instruments were set up both indoors and outdoors, and a walk-through survey of the home was conducted noting key home characteristics. Each household was given a $25 gift card to incentivize participation, once during instrument setup and once during instrument pickup. Prior to beginning the recruitment process, compliance approval was obtained from the University of Colorado Boulder’s institutional review board (Protocol 14-0734) for performing this scientific study involving human subjects.

### 2.2. Time Activity Diary

Home occupants were asked to fill out a diary of activities in which they recorded the number of hours of spent performing specific activities during the sampling period. The activities included cooking, leaving exterior doors or windows open, running air conditioning units or swamp coolers, running kitchen or bathroom exhaust fans, and noting the times when none of the occupants were home (pets could still be home).

### 2.3. Air Quality Instrumentation

Simultaneous continuous measurements of air pollutants were taken both indoors and outdoors for two to seven days at each home. Pollutants of interest were identified based on regulatory standards and public health implications, availability of reference scientific data to validate our measurements, availability of low-cost instruments, and budget constraints. Black carbon and nitrogen dioxide were not sampled for the 2016 deployment period but were added on during the 2017 sampling campaign to capture additional data on specific traffic-related air pollutants. Figure S1 illustrates the instrumentation rigs used for indoor and outdoor sampling. No data were collected in detail regarding surrounding environment’s vegetation types and crown diameters. However, outdoor monitors were positioned so that they were at least 3 m away horizontally from any immediate obstruction such as building walls or bushes, 10 m away from drip lines of trees and 1.5 m above ground level, attempting to follow the EPA Probe and Monitoring Path Siting Criteria for Ambient Air Quality Monitoring (40 CFR Appendix E to Part 58) [39] as far as practically possible. Indoor monitors were placed as far as possible, away from windows, fireplaces, kitchen stoves, water heaters, fans and furnaces and were positioned such that the home occupants felt comfortable getting around the instrument rigs.

To establish significant confidence in our measurements from the low-cost instruments, co-location experiments were performed with federal reference monitors from the Colorado Department of Public Health and Environment for instrument calibration as well as data validation. Figure S2 depicts the timeline of sampling and co-location periods.

#### 2.3.1. Particulate Matter

Number concentrations of fine particulate matter were measured using Dylos monitors (Model 1700, Dylos Corporation, Riverside, CA, USA). Dylos-1700 is a laser-based optical particle counter that reports the number concentrations (particles per cubic centimeter, #/cm^3^) of PM in two size bins: small particles with diameters 0.5 microns and above, 0.5 microns being the lower detection limit of the instrument, and large particles with diameters 2.5 microns and above. The difference between these two reported values represents the number concentration between 0.5 and 2.5 microns in particles per cubic feet (referred to from here on as PN_0.5–2.5_).

#### 2.3.2. Black Carbon

Real-time black carbon (BC) data were collected using aethalometers (MicroAeth® AE51; AethLabs, San Francisco, CA, USA), which are based on optical measurement of light transmission through a 3 mm spot created on a white filter strip containing insert of T60 Teflon-coated borosilicate glass fiber filter material. Each aethalometer was loaded with a fresh filter strip before sampling. Sampling frequency was set to 60 s and a flow rate setting of 50 mL/min was chosen to account for the expectation of high filter loading rates for near-road outdoor sampling since most of the study homes were near highly trafficked roads (distance to the closest major road <200 m). Preliminary evaluation of time series data after sampling showed that the data had significant noise and low signal-to-noise ratio. The optimized noise-reduction algorithm developed by the United States Environmental Protection Agency [40] was used for smoothing of the time series data.

#### 2.3.3. Carbon Monoxide

Custom-built open-source low-cost instruments were used for the measurement of CO with electrochemical sensors, temperature, and relative humidity (Y-Pods, Hannigan Lab, University of Colorado Boulder [41]). Y-Pods are based on an Arduino platform [42] with on-device data-logging capacity on a micro-SD memory card. Co-locations with reference instruments were crucial for the conversion of the raw voltage signals to meaningful pollutant concentrations. A post-processing algorithm was used for assimilating the co-location-generated calibration curves with the field data [43]. Co-location experiments were performed in both 2016 and 2017 at the Colorado Department of Public Health and Environment’s Continuous Air Monitoring Program station in downtown Denver [44] for calibrating the CO sensors of Y-Pods. Besides CO, Y-Pods also collected data on temperature and relative humidity (data presented in the Supplementary Table S1).

#### 2.3.4. Nitrogen Dioxide

Passive samplers from Ogawa Inc. (Ogawa, Pompano Beach, Florida, USA) were used for both indoor and outdoor measurement of time-weighted average concentrations of NO_2_. The passive sampler consists of a pre-coated sample collection paper pad coated with Triethanolamine placed inside a Teflon sampler body and secured in place by diffusion end caps. The samplers retrieved from the field were shipped to Ogawa Inc. for lab analysis along with field blanks for blank correction. Proper sampler storage, sampler preparation, and sampling protocols were followed according to the specifications from the manufacturer. All the samples were blank corrected.

#### 2.3.5. Instrument Rig Locations

All the indoor instruments were set up such that they were at least two feet away from any wall, at a height of 1.5 m, in a room free of combustion appliances, at least 1.5 m away from any fireplace or woodstove, and not immediately adjacent to an exterior window. Home occupants were asked not to open windows during sampling in the room where the indoor instruments were setup. Outdoor instruments were set up between 0.6 and 3 m away from the closest exterior wall of the homes.

### 2.4. Data Filtration

One-minute time-resolved data of PN_0.5–2.5_, BC, and CO concentrations in each home indicated that indoor pollutant concentrations could spike for short periods by orders of magnitude above the outdoor level during indoor source-induced events like cooking (the indoor source events were verified with the time activity diaries filled out by the participants). Several past studies have found similar scenarios and have found that indoor PM_2.5_ concentrations are generally higher than outdoor levels [45,46,47].

**Figure 1:**
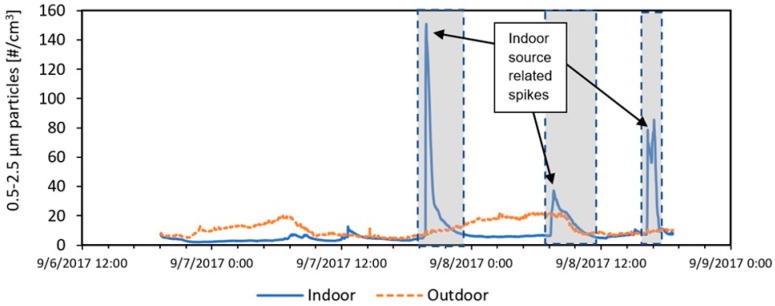
Example from one study home showing the data filtration process. Raw time series data from the shaded regions were removed, and the remainder of the time series was treated as “filtered data”.

In order to focus our analysis on impacts from outdoor pollutants and disregard indoor source effects, data filtration was performed similar to a past study from Allen et al. (2003) [46], as shown in Figure 1, in which indoor concentration spikes due to reported indoor activities such as cooking were removed from the dataset. The remainder of the data were then analyzed as a filtered set of data (referred to as “filtered data” from here on). Table S2 in the supplementary section provides comparisons between indoor and outdoor pollutants before and after data filtration.

For NO_2_ data, the homes with gas stoves were not included in the assessment of the impact of outdoor pollutants because both past studies [48,49,50] and our data showed that homes with gas stoves had significantly higher indoor NO_2_ concentrations.

### 2.5. Wildfire Impacts

Remote sensing data on wildfire smoke plume PM mass density from the National Oceanic and Atmospheric Administration’s Hazard Mapping System (NOAA HMS) [51] were used for categorizing the mesoscale impact level of wildfire smoke plumes on the study region. The plume PM density from the NOAA HMS is based on area coverage of wildfire-related smoke plume aerosols optically detected by NOAA satellites, and the satellite imagery is visually analyzed by experts each day. The plume was categorized into three levels of smoke-related PM densities [52]: the low category corresponds to the smoke PM density ≤6 µg/m^3^, the medium category corresponds to smoke PM density of ≤15 µg/m^3^ and high category corresponds to smoke PM density ≤27 µg/m^3^.

### 2.6. Distance from the Closest Major Road

The distance of the study homes from the closest major road was evaluated using the Online Transportation Information System database maintained by the Colorado Department of Transportation [53], where a major road is defined as a road with annual average daily traffic of greater than 10,000 vehicles [54,55,56]. Homes were grouped according to a distance of <100 m, 100 to 200 m, and >200 m from a major road based on the evidence from past studies that traffic-related air pollutant concentrations drop to background levels after moving away from a major road by between 100 and 200 m [57,58].

### 2.7. Data Analysis

Pollutant concentration distributions were investigated using a combination of parametric and non-parametric approaches. All pollutant concentrations were not normally or log-normally distributed (Anderson–Darling, *p* < 0.5). Hence, the non-parametric Kruskal-Wallis (K-W) and Wilcoxon Mann–Whitney tests were used for a statistical comparison of median pollutant concentrations. Correlations between variables are reported as Pearson’s correlation coefficient (r) unless otherwise stated. All statistical analyses were performed using R programming language (Version 3.4.4, R Core Team, Vienna, Austria).

## 3. Results

### 3.1. Study Household Characteristics

**Figure 2:**
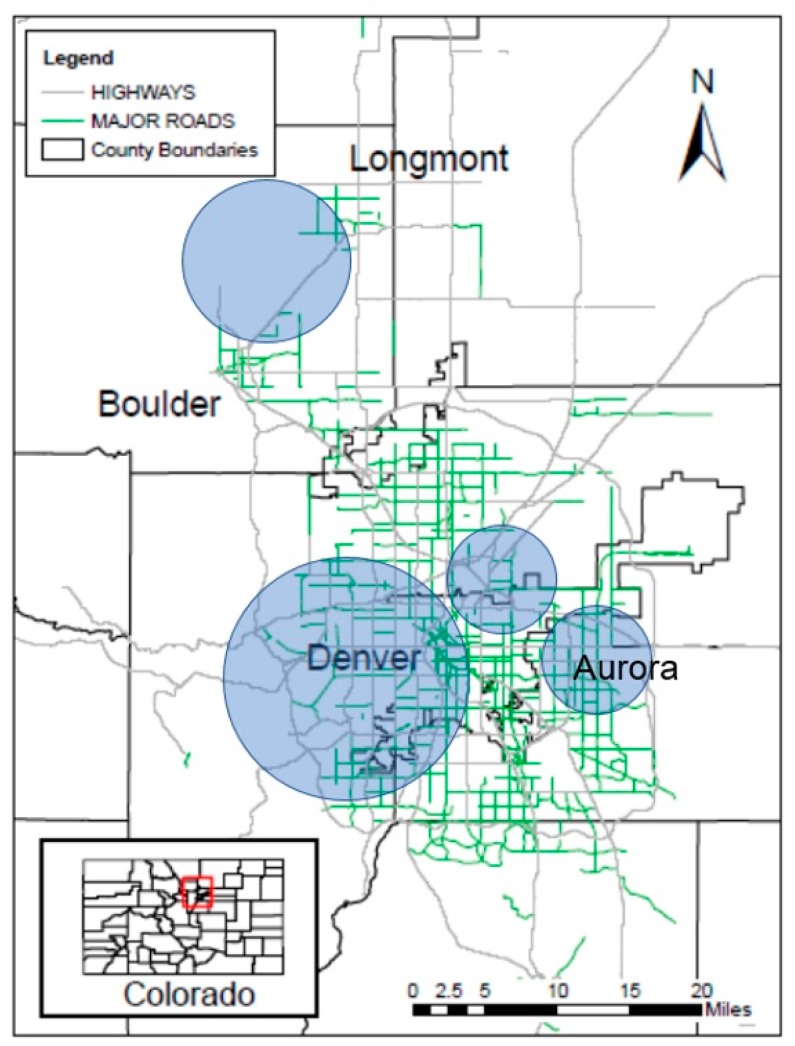
Map of the study region. Shaded circles indicate the areas of recruited homes and sizes of the circles indicate the approximate relative proportions of the number of homes in each area (Aurora: *N* = 4, Boulder/Longmont: *N* = 9, West Denver: *N* = 11; Central/North Denver: *N* = 4).

Twenty-eight homes were included in the study, located in Boulder, Longmont, and Denver Colorado (Figure 2). Because of the differences observed in household demographics, the recruited home locations were separated into four major regions: Aurora (East Denver), Boulder/Longmont, West Denver, and Central/North Denver. Five homes were specially built low-income homes for improved energy efficiency by Boulder Housing Partners with airtight construction, rooftop solar panels, all-electric air heating, and water heating systems. There were two different kinds of mechanical ventilation systems observed in these five homes: three of these homes also had heat recovery ventilation, which were intermittently and automatically operated for brief periods of time each day with timer switches, and the other two homes had continuously running exhaust fans. One home was tested both years for a sample size of 10 homes during 2016 and 19 homes during the 2017 wildfire seasons. Sixteen of the homes had at least one window left open for more than twelve hours during the sampling period. Five of the homes had gas stoves, whereas 23 homes had electric stoves. Eight of the homes had stove hoods that exhausted outdoors, 13 were of the indoor recirculating type and seven homes did not have any kind of stove hood present. Table S1 summarizes key home characteristics.

### 3.2. Wildfire Impacts on Outdoor Particulate Matter in the Study Region

**Figure 3:**
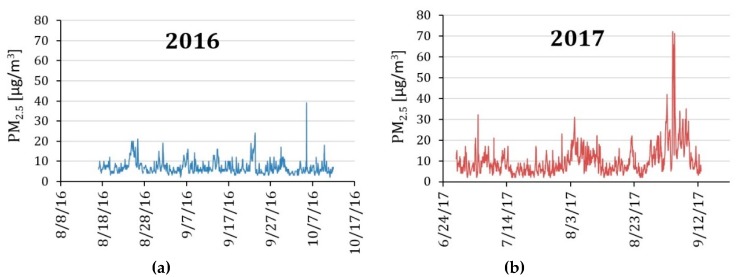
Time series data of particulate matter (PM_2.5_) concentration measurements made at Colorado Department of Public Health and Environment’s Continuous Air Monitoring Program (CAMP) station in Downtown Denver during our instrument deployment periods in (**a**) 2016 and (**b**) 2017.

PM_2.5_ mass concentrations recorded at Colorado Department of Public Health and Environment’s Continuous Air Monitoring Program (CAMP) station in downtown Denver is illustrated as a time series plot in Figure 3 for the sampling periods when we deployed the air monitoring instruments in the study homes.

There were a few spikes of elevated PM_2.5_ concentrations, particularly towards the end of the 2017 sampling period. These periods coincided with several days of reduced visibility due to long-range wildfire plumes from Canada and the Western regions of the United States, which was confirmed using NOAA HMS satellite imagery for the time periods corresponding to the pronounced spikes. The summer of 2016, however, had comparatively low outdoor PM_2.5_ during our field deployment period. Both 2016 and 2017 deployment periods had several days of outdoor PM_2.5_ levels above the primary one-year National Ambient Air Quality Standard for PM_2.5_ of 12 µg/m^3^. Typically, the Denver metro area complies with the PM_2.5_ standard [59].

**Figure 4:**
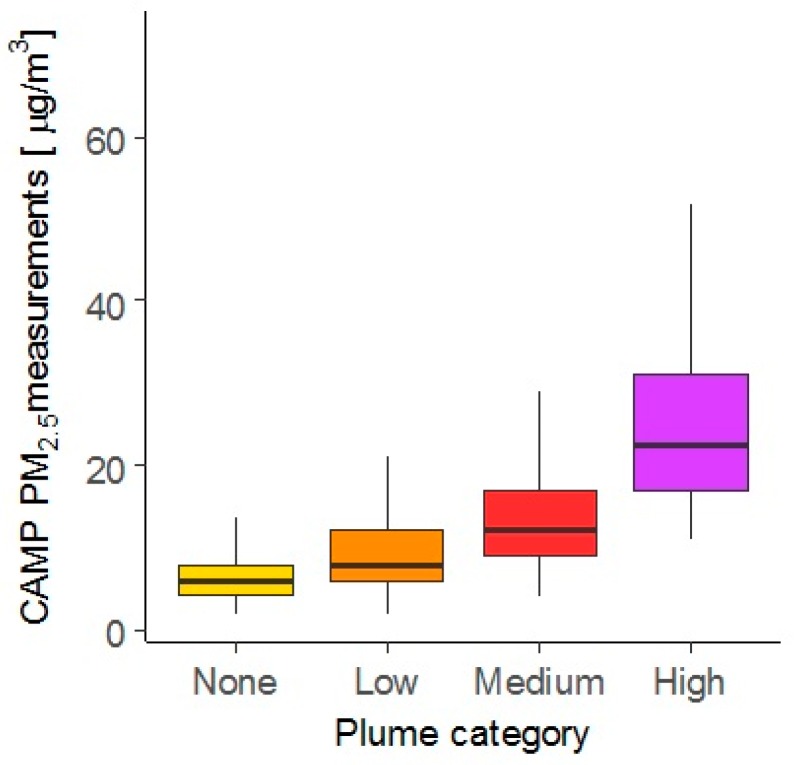
Boxplots (without outliers) showing plume categories and the corresponding PM_2.5_ concentration measurements made at Colorado Department of Public Health and Environment (CDPHE)’s Continuous Air Monitoring Program (CAMP) air monitoring station in Denver. Data were pooled together from the deployment periods from 17 August 2016 to 10 October 2016 and from 28 June 2017 to 12 September 2017. (Kruskal-Wallis (K-W) test: *p* < 0.01.)

Study homes were categorized according to the wildfire-related plume cover density in the study region during the instrument deployment period. Categorization of plume cover over the study region corresponded well with the ground level measurements taken at the CAMP air monitoring station in Denver (Figure 4). The CAMP hourly measurements of PM_2.5_ monotonically increased from a median concentration of 6 to 8 (+33%), 8 to 12 (+50%) and 12 to 23 µg/m^3^ (+92%) between the plume categories None, Low, Medium, and High, respectively (K-W test: *p* < 0.05). The median difference between the High and None categories was 17 µg/m^3^, which was 2.8 times the background level (representative of traffic and other local emission sources) of 6 µg/m^3^.

### 3.3. Indoor and Outdoor Pollutant Measurements

#### 3.3.1. Data Capture

The number of homes for which data were available for each measured pollutant varied due to data recovery issues; some instruments suffered from power failure, sensor malfunction, or some other technical issue that prevented data capture. Out of the 29 times when our instruments were deployed to record data (measurement in one home was taken both in 2016 and 2017, resulting in our dataset of 29 unique measurements done in 28 different homes), the total number of homes for which data were available both indoors and outdoors ranged between 17 and 27 homes. BC and NO_2_ measurements were only added in 2017, whereas the rest of the pollutants were measured for both 2016 and 2017 periods. Table S3 summarizes the measured pollutant concentrations indoors and outdoors from raw datasets.

#### 3.3.2. Particulate Matter

Raw time series data of indoor and outdoor PN_0.5–2.5_ captured by the Dylos monitors showed that outdoor concentrations were mostly higher than indoor concentrations except when there were spikes in the indoor concentrations caused by indoor activities like cooking. This was true even in the absence of wildfire plumes. The fraction of sampling times when outdoor concentrations were higher than indoors during each field deployment was 59% of the cumulative total sampling time in all the homes.

**Figure 5:**
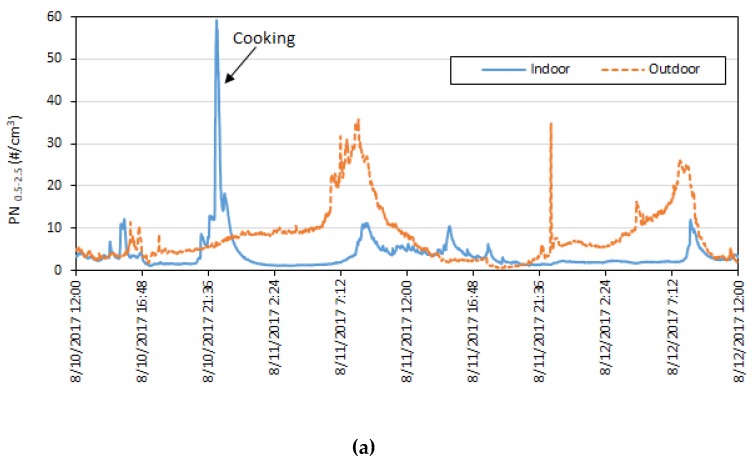

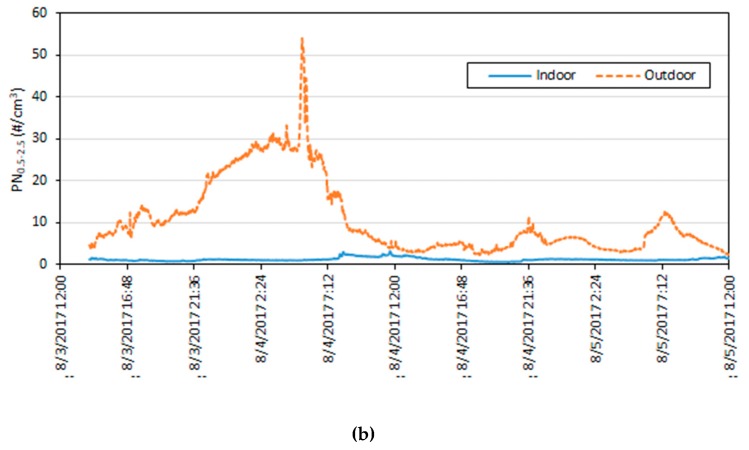
Indoor and outdoor time series PN_0.5–2.5_ data from two of the homes tested ((**a**): Home T442, (**b**): Home T450).

Figure 5 shows an example of the time series of PN_0.5–2.5_ concentrations from two of the homes. In Figure 5a, one cooking-related spike can clearly be seen in the indoor PN_0.5–2.5_ concentration. The field deployment period for this home coincided with a plume cover event of medium density, which explains the distinct rise in outdoor PN_0.5–2.5_ concentrations compared to background levels, lasting for roughly six to seven hours at a time. The occupants of this home kept a window open at all times during testing. This pattern was also seen in other homes and the profiles of indoor concentrations were seen to follow the outdoor concentration spikes in most cases.

The home shown in Figure 5b had no significant indoor sources during sampling. All the windows in this home were closed throughout the sampling period. The significant elevation of outdoor PN_0.5–2.5_ concentration period in the first half of the sampling period also coincided with a plume cover event of medium density over the study region.

#### 3.3.3. Black Carbon

**Figure 6:**
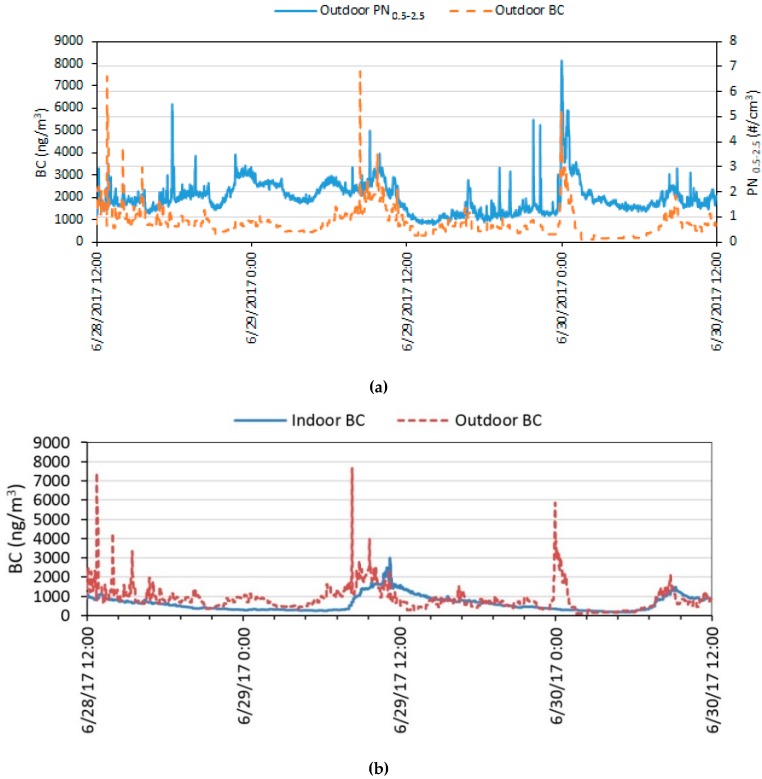
Time series profiles of black carbon from one of the test homes (Home ID: T109). (**a**) Compares the outdoor concentration profiles of black carbon (BC) and PN_0.5–2.5_, and (**b**) compares the indoor and outdoor BC profiles.

Black carbon was measured only during the 2017 campaign. Time series of outdoor and indoor BC time series profiles (raw datasets) from one of the homes tested with home identification number (Home ID) T109 are shown in Figure 6. Table S2 shows that Spearman’s rank correlation between indoor and outdoor BC were positively correlated (r_s_ = 0.51, *p* < 0.000). Outdoor BC was also correlated with outdoor PN_0.5–2.5_ (r_s_ = 0.56, *p* < 0.000). Very few homes had indoor source-related spikes, suggesting that most of the BC in homes originated outdoors. A past study has also shown that outdoor vehicular traffic emissions directly affect indoor BC levels despite windows remaining closed at all times [60]. Outdoor BC concentrations were higher than indoors 66% of the total sampling time in all homes.

The effect of window opening on BC concentrations can clearly be seen from Figure 6b. The outdoor BC concentration spike at 00:00 on 30 June 2017 that lasted for at least one hour had no effect on the indoor BC concentration because the windows were closed at night. During the daytime, however, the windows were left open and the indoor concentrations closely followed the outdoor profiles.

#### 3.3.4. Carbon Monoxide

The carbon monoxide levels measured in this study were low, averaging 0.69 ppm indoors and 0.20 ppm outdoors (raw dataset). A total of 12 homes had indoor and outdoor concentrations that were similar, while 16 other homes had higher indoor average levels of CO. Three of these homes with higher indoor CO also had gas stoves. Only two out of the 28 homes sampled had electric water heaters, whereas the remainder of the homes had gas water heaters with standing pilot lights. A total of 25 of the homes also had forced air heating systems using natural gas as fuel.

#### 3.3.5. Nitrogen Dioxide

**Figure 7:**
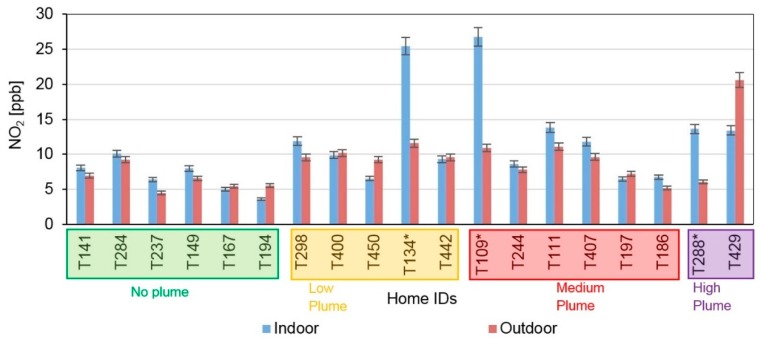
NO_2_ concentrations measured in 2017 in all homes (*n* = 19). Home IDs with asterisk represent homes with gas stoves. Error bars indicate sampler uncertainty. Homes are ordered from left to right with increasing wildfire plume densities during sampling.

Nitrogen dioxide was measured only during the 2017 campaign. Three homes with gas stoves had significantly high indoor NO_2_ compared to outdoors (Figure 7; T134, T109, T288). The median indoor concentration of NO_2_ was roughly three times higher, and the indoor/outdoor NO_2_ ratio was 2.3 in these homes with gas stoves compared to 0.98 in the homes without gas stoves, a result not likely due to chance (K-W test, *p* = 0.007: Table S3). These homes were not included in the subsequent data analysis of outdoor NO_2_ infiltration. In the rest of the homes, indoor and outdoor concentrations of NO_2_ were comparable to each other and not impacted by wildfire plumes. In all cases, the concentrations of NO_2_ were lower than the National Ambient Air Quality Standard (NAAQS) of 53 ppb.

### 3.4. Impacts of Road Proximity

**Figure 8:**
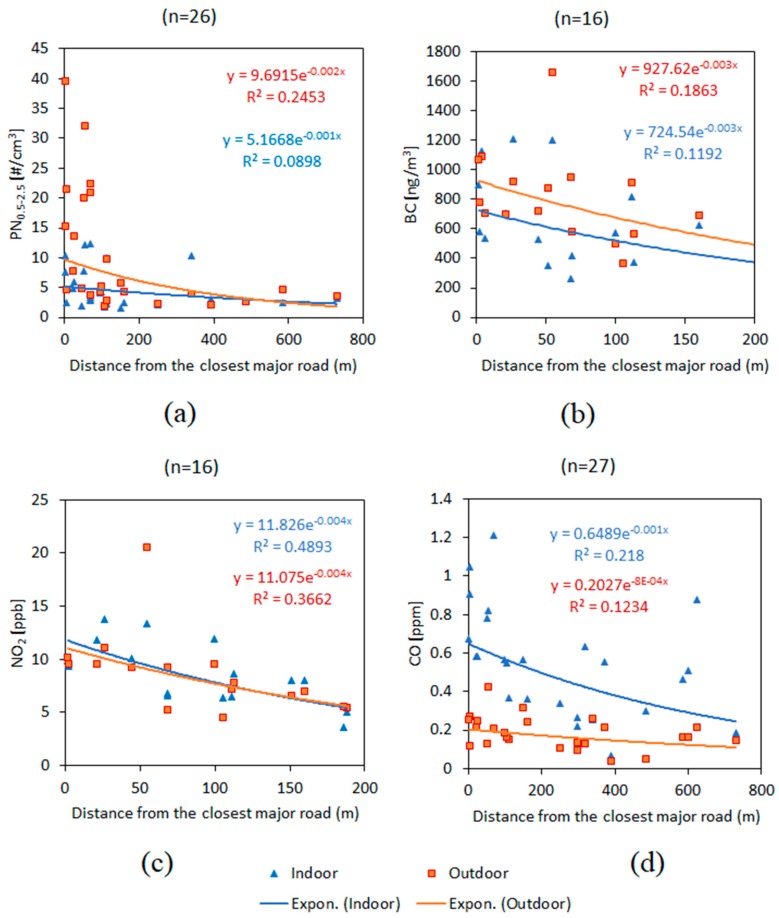
Pollutant concentrations as a function of distance from the closest major road (exponential curve-fitting) for (**a**) PN_0.5–2.5_, (**b**) BC, (**c**) NO_2_, (**d**) CO. This dataset does not include the three homes with gas stoves.

Outdoor pollutant concentrations monotonically decreased with increasing distance from the closest major road, a finding in good agreement with a number of previous studies [61,62,63]. It was noted in our study, however, that the geometric means of PN_0.5–2.5_, BC, and NO_2_ outdoor concentrations had more significant decline than CO with increasing distance from the major road (Figure 8). Exponential curve-fitting was used in Figure 8 for comparability with past studies and exponential nature of general air pollutant dispersion phenomenon [64,65,66,67]. Outdoor and indoor NO_2_ concentrations rose monotonically and almost identically with respect to the distance from the closest major road. Indoor median PN_0.5–2.5_ was 15% higher in homes located closer to the roads.

### 3.5. Filtered Indoor and Outdoor Measurements

**Figure 9:**
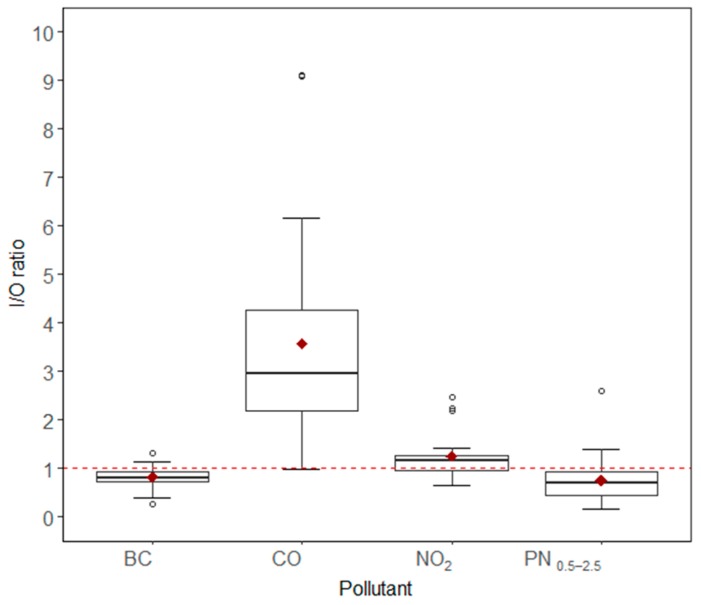
Tukey boxplot showing indoor/outdoor ratios of all pollutants calculated from filtered dataset. Lower and upper bounds of the boxplot represent first quartile and third quartiles (Q1 and Q3, respectively; middle bar inside the box represents the median, middle diamond inside the box represents the mean value, lower and upper whisker limits indicate Q1 − 1.5x (inter-quartile range) and Q3 + 1.5x (inter-quartile range), respectively, and the dots outside the whisker limits indicate outliers.

After filtering the datasets for indoor pollution-generating activity spikes, the median indoor/outdoor (I/O) ratios of CO and NO_2_ were greater than one (Figure 9), whereas for PN_0.5–2.5_, and BC they were less than one, which was also seen with the raw datasets. Indoor CO concentrations in the homes were two to four times the outdoor concentrations. Indoor median CO concentrations were close to 1 ppm in most homes (raw data) and well below the eight-hour NAAQS for CO of 9 ppm. Indoor CO concentrations in four homes were often elevated above 1 ppm and the median indoor CO concentration for these homes ranged from 1.5 to 3.1 ppm. Three of these homes had gas stoves, while the fourth did not. Eleven homes had elevated levels of CO (three to five times higher indoors compared to outdoors), even when indoor source-related spikes were filtered out as reported in the time-activity diaries.

**Figure 10:**
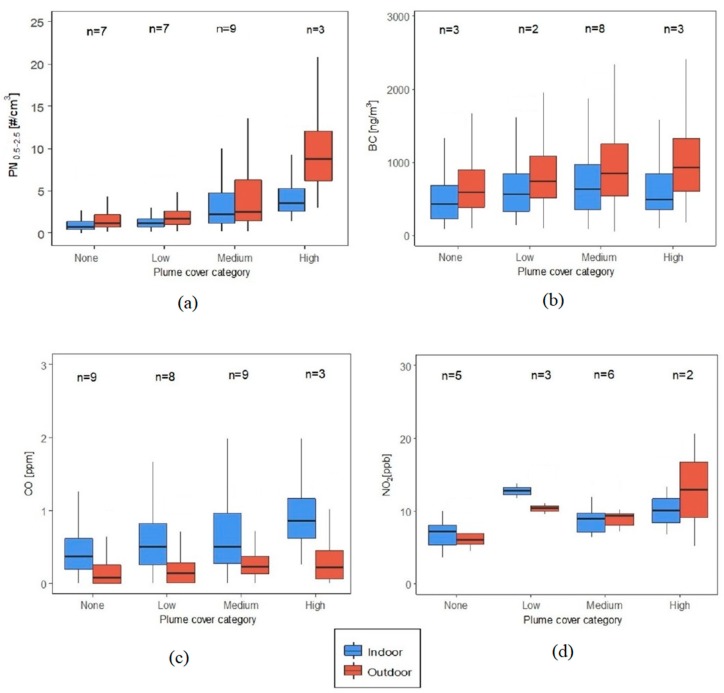
Indoor and outdoor pollutant concentration distributions (not showing outliers) according to wildfire plume cover (filtered datasets) for (**a**) PN_0.5-2.5_, (**b**) BC, (**c**) CO and (**d**) NO_2_. The indoor and outdoor concentrations between all plume categories were significantly different for all pollutants except NO_2_ (K-W test at α = 0.05).

Wildfire plumes caused a significant rise in the median outdoor as well as indoor concentrations of most pollutants (Figure 10). Outdoor median PN_0.5–2.5_ was 6.4 times higher and indoor median PN_0.5–2.5_ was 3.6 times higher during the High plume cover compared to the times with no plume cover. The I/O ratio was the highest for CO in the High plume category and was 35% higher than the None category, indicating some CO was due to the smoke plume. In the absence of any wildfire plumes, outdoor median concentrations of PN_0.5–2.5_ and BC were 1.6 and 1.4 times higher than indoors, respectively, which can mostly be attributed to traffic-related emissions in the absence of other significant local outdoor and indoor sources. Wildfire plumes did not affect NO_2_.

Table S4 in the supplementary section presents comparisons between indoor and outdoor medians and ranges for various measured pollutant concentrations (from the filtered dataset) classified according to house characteristics. The location of the homes significantly affected pollutant concentrations and I/O ratios. Homes built in the Aurora region had the lowest median I/O ratios for PN_0.5–2.5_ and BC, whereas I/O for CO was highest in Aurora. Homes in Central/North Denver regions had the highest outdoor median concentrations of PN_0.5–2.5_ and BC.

With respect to mechanical ventilation (MV), the median I/O ratio of PN_0.5–2.5_ and BC were higher by 18% and 4%, respectively, when MV was present, indicating that the outdoor air pollution was brought indoors through ventilation supply air and not adequately filtered out. The MV systems only had low efficiency filters designed primarily to protect the equipment and not intended to provide clean air supply to the conditioned zone. The median I/O ratio of CO was lower in homes with MV by 3% indicating the ventilation removed the indoor CO.

The impact of gas stoves was seen even in the filtered datasets. Although homes with gas stoves were not included in the NO_2_ dataset, the rest of the pollutant datasets still included the homes with gas stoves. The I/O ratios for CO were significantly higher (+57%) in homes with gas stoves.

The type of kitchen stove hood also had a significant impact on indoor pollutant concentrations. In the filtered datasets, the homes with exhaust type stove hoods had PN_0.5–2.5_ I/O ratios 49% less than the homes with recirculating hoods and 55% less than the homes with no stove hoods installed.

**Figure 11:**
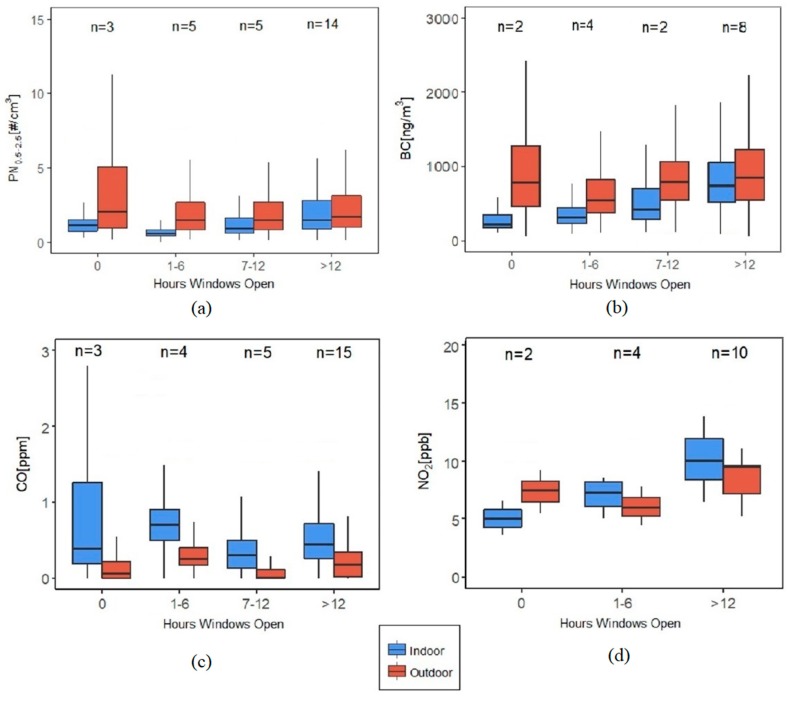
Indoor and outdoor pollutant concentration distributions (not showing outliers) according to the hours of window opening (filtered dataset) for (**a**) PN_0.5-2.5_, (**b**) BC, (**c**) CO and (**d**) NO_2_. The indoor and outdoor mean concentrations were significantly different across all window opening intervals for all pollutants except for NO_2_ (K-W test at α = 0.05).

Home occupants relied on window opening for thermal comfort, mainly because they did not have air-conditioning, resulting in almost instantaneous transfer of pollutants between outdoors and indoors. The time-activity diary revealed that most homes (25 out of 28) had at least one window open for at least one hour a day. Indoor median BC concentration had a monotonic rise with the number of hours of at least one window open in the house. However, a similar rise was not seen with PN_0.5–2.5_ (Figure 11). Window opening also had a significant impact on the I/O ratio of CO, with the highest I/O ratio for the homes that had all the windows closed throughout the sampling period, which was roughly three times higher than having the window open for even a small fraction of the time.

## 4. Discussion

The main objective of this study was to investigate the impact of outdoor pollution on indoor air quality during wildfire seasons in Colorado. Data filtering was performed to remove the spikes in indoor concentrations of all the pollutants from indoor sources or activities, which were mostly cooking related. Spearman’s rank correlations (r) were also calculated between indoor and outdoor concentrations for both raw and filtered data. Filtering the data changed the concentration distributions of all indoor pollutants. The median indoor concentrations of PN_0.5–2.5_, CO, and BC, due to data filtration were reduced by 16%, 7%, and 4% respectively; differences were statistically significant for PN_0.5–2.5_ and CO, suggesting that cooking indoors was a significant source of PN_0.5–2.5_ and CO. Table S2 in the supplementary section summarizes the comparison between raw and filtered datasets. Table S4 presents the I/O ratios, based on the filtered dataset.

While the indoor/outdoor ratio was used in this study as an effective metric for a comparison of pollutant concentrations between indoors and outdoors, it should be noted that the I/O ratio can decrease not just because of the decrease in indoor concentration, but also due to an increase in outdoor concentration (or a combination of both). Hence, it is important to also refer to the median indoor and outdoor concentrations to help elucidate associations. Median values are reported here instead of arithmetic means because of their robustness towards outliers. The I/O ratios and concentrations of all pollutants were positively skewed but not log-normally distributed (A-D test: *p* < 0.000).

Our results showed that 11 homes had indoor concentrations of CO three to five times higher than outdoors even when indoor source-related spikes (as determined from time activity diaries) were filtered out from our dataset. One possible explanation to this observation is the effect of standing pilot lights in combustion devices like gas water heaters and fireplaces. All the homes we studied had CO alarms installed properly, and were in working condition, and all the levels measured were well below the one-hour NAAQS for CO of 35 ppm, so the occupants were not in immediate danger of acute CO poisoning during our sampling. The health impacts of chronic exposure to lower levels of CO are less clear [68]. Potential links between ambient levels of CO and behavioral abnormalities in children and effects of subclinical exposure on the brain during development have been discussed [69]. A study of elderly men showed increased carboxyhemoglobin levels in subjects who used gas for cooking [70]. More research should be done to determine whether these results are more widespread in homes so that better venting of combustion appliances can be addressed by introducing appropriate building codes that require mandatory outdoor venting of gas stoves.

Data from our study are consistent with previous studies in many respects. Previous studies of indoor and outdoor particulate matter in buildings in the front range of Colorado show that concentrations depend on location, and that concentrations are typically higher in Denver compared to less urban cities such as Boulder [71]. A study of 15 homes in Boulder/Ft. Collins measured PM_2.5_ concentrations that were highest during the summer. Our measurements of NO_2_ outdoor concentrations from the raw dataset are also in agreement with a study using the same sampler that shows an exponential rise in outdoor NO_2_ with decreasing distance to a major road [72]. Our finding of indoor NO_2_ concentrations being two to three times higher in homes with gas stoves is also consistent with past studies [73,74]. In a previous study, BC concentration ranged from 3.4 to 10 µg/m^3^ at a distance of 30 m from an interstate highway, and closely tracked the concentration profiles of particulate matter and CO [75]. These levels are higher than what was measured in this study: we measured peaks of 1.2–1.6 µg/m^3^.

As many previous studies have found, indoor sources cause peaks in indoor pollutant concentrations. However, traffic and wildfire pollutants add to the levels observed indoors. Filtering the dataset to remove obvious indoor source-related spikes in concentrations of the pollutants gave a better picture of how building shells generally interact with the outer environment. The correlations between indoor and outdoor pollutant concentrations were higher when the dataset was filtered (Table S2), indicating that filtering achieved our objective of reducing the data to reflect the outdoor contribution to indoor concentrations. The building envelope is intuitively thought of as the protective layer between the indoor environment and outdoor air pollution. Our data suggest that even without indoor sources, median indoor concentrations of CO can be elevated by up to three-fold during when wildfire plumes are impacting local outdoor air and by four-fold due to roadway proximity although indoor concentrations of PN_0.5–2.5_ and BC were found to be less than or close to outdoor concentrations during wildfire plumes as well as close proximity to the roads (Table S4). Past studies have identified that even when windows remain closed the indoor PM levels are directly affected to a measurable extent by outdoor PM concentrations. Window opening behavior in response to temperature changes add to the introduction of outdoor PM indoors, especially in low-income communities who have no easy access to air conditioning and indoor air filtration technologies [76,77].

There were also a number of limitations in our study. Due to the nature of the study, very strict recruitment criteria could not be implemented, for example homes with the exact same layout, or number of exterior doors and windows. Hence, homes recruited were randomized in terms of the number and sizes of windows, building materials, etc. Despite the attempt to collect outdoor meteorological data from homes, the data retrieval rate from Y-Pods was very low for the weather station connected to the Y-Pods. Hence, no statistical comparisons could be made between pollutant I/O ratios and wind speeds. Also, the study was conducted during the summer season and the majority of the home occupants resorted to opening windows for thermal comfort. This meant that we could not associate the pollutant concentrations indoors to the air tightness of the building shell structure. Natural ventilation rates have been shown to have an impact on indoor fine PM deposition rate, which can result in different indoor exposure levels in case occupants have all windows closed during wildfire plume events [78]. Hence, future studies can incorporate studying the impact of the leakage of building shell structure on pollutant I/O ratios in low-income households.

Low-income households in the U.S. can benefit from this study in a number of ways with respect to the improvement in public health. High indoor concentration spikes of pollutants associated with indoor sources like cooking are difficult to avoid in real-life, thus to reduce exposure better strategies can be implemented to ensure protection from outdoor air pollution in low-income homes, such as installing cooling devices so that windows can remain closed during pollution episodes and distributing air cleaners on especially polluted days. As seen from Tables S3 and S4, I/O ratios for PN_0.5–2.5_ were highest for homes without kitchen stove hoods, followed by homes with recirculating stove hoods when compared to exhaust-type stove hoods, a finding in agreement with another study [79]. Indoor source-related emissions should still be addressed with engineering approaches such as source control (stove exhaust hoods) or other strategies to reduce air pollutant exposure. The I/O ratio for temperature is almost always greater than one and the I/O ratio for relative humidity is almost always less than or close to one in all homes (Table S1). This means that air tends to get drier as it infiltrates indoors into the residential indoor spaces. Logically speaking, this tendency can increase the ultrafine fraction of suspended particulate matter after infiltrating as the volatile part of the PM evaporates under the drier conditions, thus reducing the PM aerodynamic diameter. Homes close to major roads and highways are particularly more vulnerable to having more ultrafine particulate matter infiltration, hence, resulting in greater public health concerns. For new housing developments close to the highways, ventilation options should be carefully analyzed to minimize the introduction of traffic-related PM to the indoor air by facing ventilation air intakes on the sides of the buildings away from the highways [80] and utilizing filtration. In addition, smart low-cost sensor technologies have a huge potential to provide greater control over the active ventilation of residential spaces. The reduction in occupant exposure to outdoor air pollution by collective knowledge and the careful use of ventilation techniques will ultimately result in better public health of low-income communities who are vulnerable to the health risk impacts of outdoor air pollution.

## 5. Conclusions

This study showed that outdoor air pollution related to traffic emissions and wildfires significantly increased the indoor air pollutant concentrations due to infiltration and natural ventilation in the 28 low-income homes that were sampled. Wildfires increased the PN_0.5–2.5_ indoors by almost four times, and BC by almost two times, compared to when there were no wildfires. Proximity to roadways influenced indoor concentrations of BC, CO, and NO_2_, significantly elevating the concentrations in homes closest to roadways compared to those more than 200 m away. Among the various pollutants measured, the I/O ratio of CO was found to be consistently two to three times higher than other pollutants measured. Regarding the factors affecting indoor pollutant concentrations, window opening significantly increased BC concentrations, but decreased CO concentrations. Indoor NO_2_ concentrations were found to be over two times higher compared to outdoors in the homes with gas stoves. Homes with exhaust hoods had lower indoor pollutant concentrations, while the homes with MV systems, although few in number, were found to have consistently higher levels of the measured pollutants indoors.

## Supplementary Materials

The following are available online at https://www.mdpi.com/1660-4601/16/19/3535/s1, Figure S1: Outdoor instrument rig (Left) and Indoor Instrument rig (Middle and Right). [Legend for labels: 1 = Weather station; 2 = Dylos-1700 Monitor (outdoor instrument covered with metallic bucket); 3 = microAeth AE51 Aethalometer (outdoor instrument covered with metallic bucket); 4 = Y-pod; 5 = Ogawa NO2 passive sampler; 6 = weather-protected electrical connection point; 7 = tripod stand.], Figure S2: Schematic showing timeline of instrument deployment periods, sensor calibration periods for Y-Pods and validation periods for sensor calibrations. CO2 data were calibrated using TSI Q-Trak as the reference instrument, but the data showed poor results during validation and, hence, CO2 data were discarded, Figure S3: Example of National Oceanic and Atmospheric Administration’s Hazard Mapping System (NOAA HMS) remote sensing data showing a day with plume cover (a) and no plume cover (b) over the state of Colorado, Table S1: Cross-tabulation of indoor and outdoor temperature and relative humidity with various home characteristics (raw dataset). Total *N* = 28, Table S2: Concentration comparisons between indoor and outdoor pollutants before and after data filtration, Table S3: Pollutant concentrations indoors and outdoors from raw datasets with the corresponding p-values from K-W test on I/O ratios between categories, Table S4: Pollutant concentrations indoors and outdoors from filtered datasets with the corresponding *p*-values from K-W test on I/O ratios between categories.
