# Supplementary material for: Impact of Outdoor Air Pollution on Indoor Air Quality in Low-Income Homes during Wildfire Seasons
Source: Int J Environ Res Public Health. 2019 Sep 21;16(19):3535. doi: 10.3390/ijerph16193535 (PMC6801919; doi:10.3390/ijerph16193535)
Supplement: Supplementary file 1 [file ijerph-16-03535-s001.pdf]

**Supplementary Materials:**

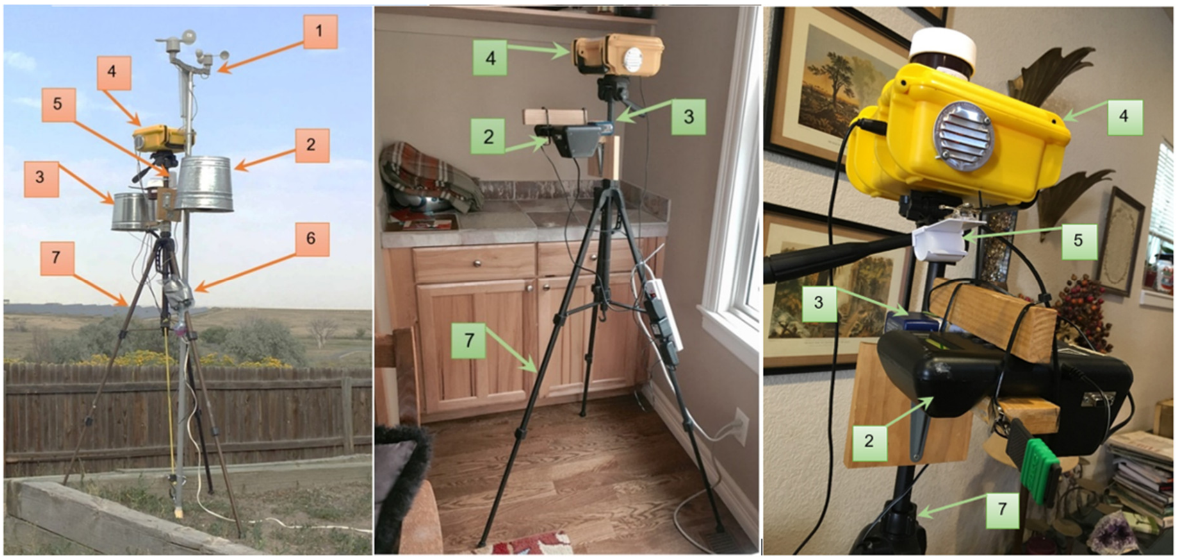


**Figure S1.** Outdoor instrument rig (Left) and Indoor Instrument rig (Middle and Right). [Legend for labels: 1 = Weather station; 2 = Dylos-1700 Monitor (outdoor instrument covered with metallic bucket); 3 = microAeth AE51 Aethalometer (outdoor instrument covered with metallic bucket); 4 = Y-pod; 5 = Ogawa $NO_2$ passive sampler; 6 = weather-protected electrical connection point; 7 = tripod stand.]

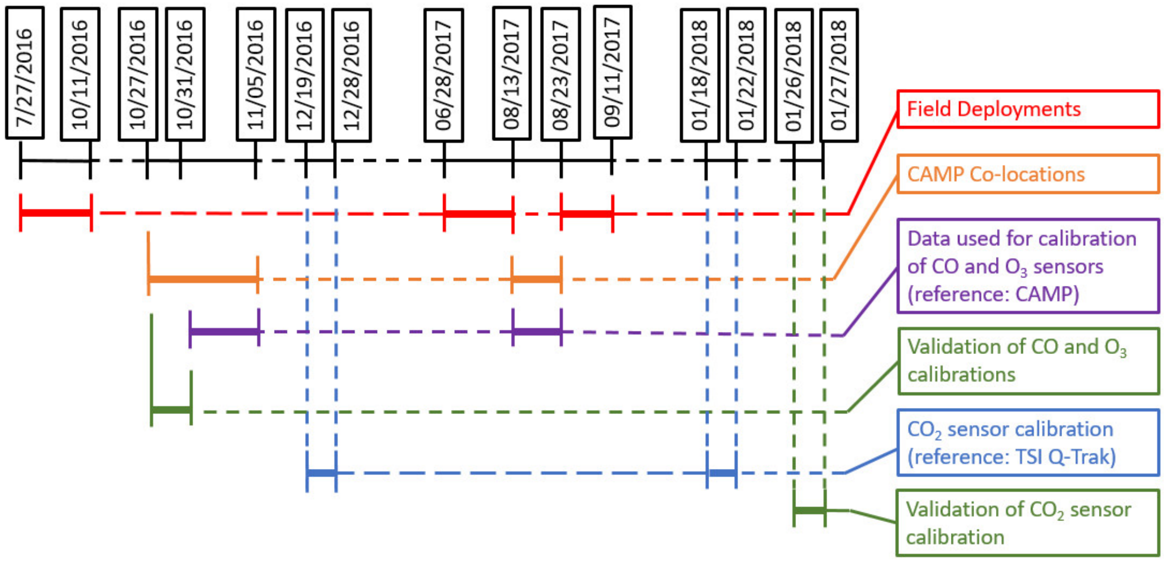


**Figure S2.** Schematic showing timeline of instrument deployment periods, sensor calibration periods for Y-Pods and validation periods for sensor calibrations. $CO_2$ data were calibrated using TSI Q-Trak as the reference instrument, but the data showed poor results during validation and, hence, $CO_2$ data were discarded.

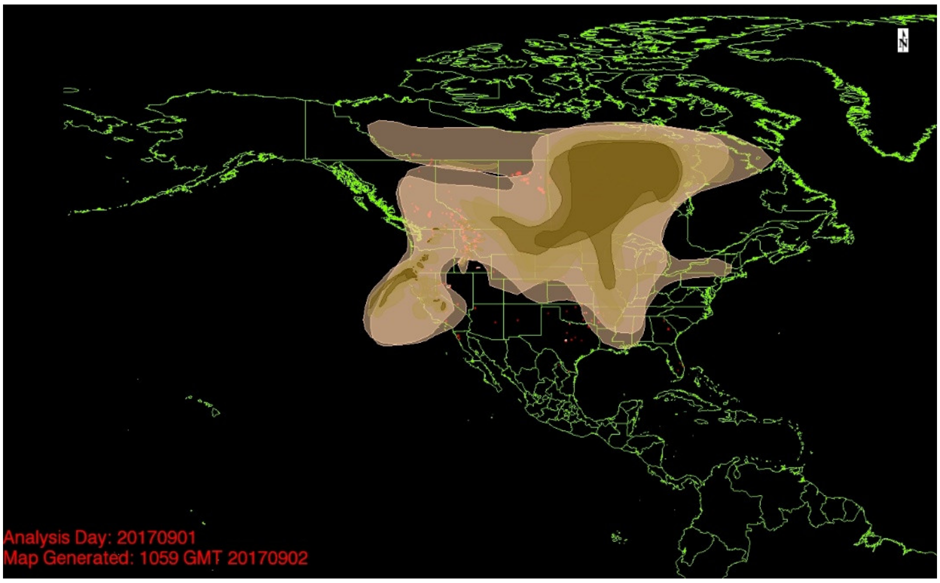


(a)

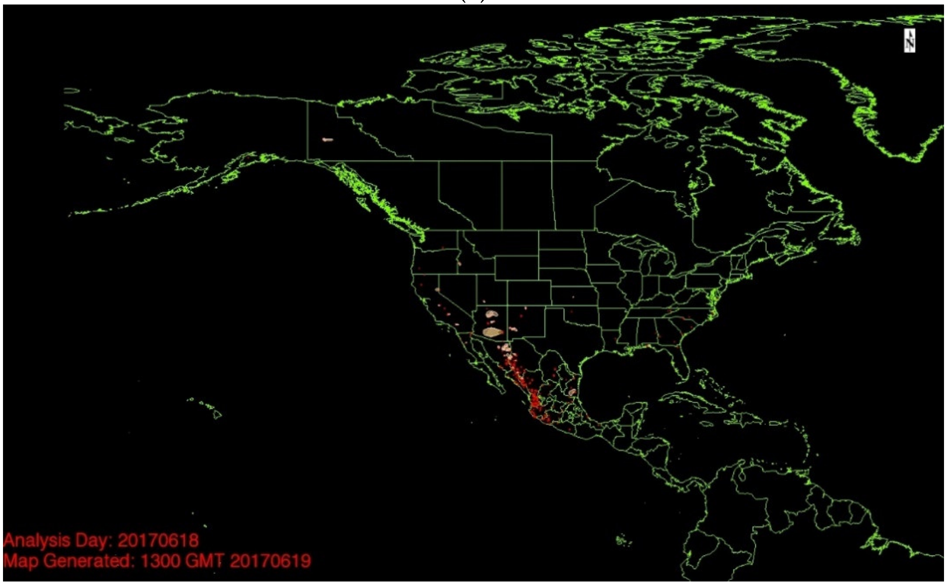


(b)

**Figure S3.** Example of National Oceanic and Atmospheric Administration's Hazard Mapping System (NOAA HMS) remote sensing data showing a day with plume cover (**a**) and no plume cover (**b**) over the state of Colorado.

**Table S1.** Cross-tabulation of indoor and outdoor temperature and relative humidity with various home characteristics (raw dataset). Total $N = 28$.

| | *n* (% of Total) | Temperature (°C) Indoor Median (Range)/Outdoor Median (Range) | Temperature (°C) I/O Ratio | Relative Humidity (%) Indoor Median (Range)/Outdoor Median (Range) | Relative Humidity (%) I/O Ratio |
|---|---|---|---|---|---|
| **Location** | | | | | |
| Aurora | 4 (14.3) | 27.3 (21.2–33.8)/ 23.5 (10.9–46.8) | 1.16 | 34.92 (25.3–46.4) / 41.6 (9.22–81.9) | 0.84 |
| Boulder/ Longmont | 9 (32.1) | 23.2 (18.9–30.2) / 16.8 (1.4–41.1) | 1.38 | 30.9 (13.7–55.0) / 35.3 (9.10–88.5) | 0.87 |
| West Denver | 11 (39.3) | 26.4 (17.1–34.0) / 21.4 (1.70–45.7) | 1.23 | 34.8 (14.6–61.2) / 37.7 (6.57–86.0) | 0.92 |
| Central/North Denver | 4 (14.3) | 26.4 (19.7–30.0) / 20.9 (9.79–44.4) | 1.26 | 34.1 (19.7–60.0) / 42.5 (7.11–84.4) | 0.80 |
| **Building Type†** | | | | | |
| BG | 5 (17.9) | 22.4 (18.9–30.16) / 12.9 (1.4–39.1) | 1.74 | 26.6 (13.7–46.7) / 29.4 (9.10–83.2) | 0.90 |
| EER | 13 (46.4) | 27.1 (17.1–34.0) / 22.2 (1.69–46.8) | 1.22 | 34.6 (14.6–55.0) / 39.9 (6.57–88.5) | 0.87 |
| Non-EER | 10 (35.7) | 25.7 (19.74–31.1) / 21.6 (9.79–44.4) | 1.19 | 40.6 (14.8–61.7) / 43.1 (7.11–86.0) | 0.94 |
| **Road Proximity** | | | | | |
| <100 m | 15 (53.6) | 25.3 (19.5–33.8) / 19.6 (1.56–44.4) | 1.29 | 34.1 (14.5–61.7) / 39.4 (7.11–86.0) | 0.86 |
| 100–200 m | 7 (25.0) | 26.7 (17.1–34.4) / 22.6 (1.69–46.8) | 1.18 | 35.2 (15.3–55.0) / 39.1 (7.54–88.5) | 0.90 |
| >200 m | 6 (21.4) | 25.4 (19.0–32.2) / 17.8 (1.4–44.8) | 1.43 | 31.1 (13.7–44.0) / 34.9 (6.57–83.5) | 0.89 |
| **Wildfire Plume Density** | | | | | |
| None | 10 (35.7) | 22.7 (17.1–34.0) / 16.5 (1.40–46.7) | 1.37 | 31.1 (13.7–55.0) / 34.5 (9.10–88.5) | 0.90 |
| Low | 6 (21.4) | 26.7 (19.8–32.0) / 22.8 (9.79–45.7) | 1.17 | 36.7 (14.7–59.8) / 40.1 (6.62–84.4) | 0.91 |
| Medium | 9 (32.1) | 26.5 (20.1–32.2) / 21.6 (10.6–44.7) | 1.23 | 36.8 (14.6–61.7) / 41.8 (6.57–86.0) | 0.88 |
| High | 3 (10.7) | 24.5 (21.2–29.5) / 21.5 (10.9–39.1) | 1.14 | 40.4 (22.9–46.7) / 35.5 (9.26–71.1) | 1.14 |
| **Mechanical Ventilation** | | | | | |
| Present | 5 (17.9) | 23.5 (19.5–34.0) / 17.8 (1.56–44.7) | 1.32 | 28.5 (14.5–47.5) / 36.9 (10.3–88.5) | 0.77 |
| Absent | 23 (82.1) | 26.0 (17.1–33.8) / 20.5 (1.40–46.8) | 1.27 | 34.7 (13.7–61.7) / 38.1 (6.57–86.0) | 0.91 |
| **Hours Windows Open** | | | | | |
| 0 | 3 (10.7) | 24.7 (19.9–28.3) / 18.4 (1.56–40.9) | 1.34 | 31.7 (16.1–44.3) / 44.8 (12.9–88.5) | 0.71 |
| 1–6 | 4 (14.3) | 26.0 (17.1–33.0) / 21.4 (1.69–46.8) | 1.21 | 34.0 (15.3–55.0) / 36.1 (7.54–84.4) | 0.94 |
| 7–12 | 5 (17.9) | 22.8 (19.0–29.5) / 16.8 (2.64–41.5) | 1.36 | 32.6 (13.7–53.9) / 36.4 (9.10–83.5) | 0.89 |
| >12 | 16 (57.1) | 26.2 (19.0–34.0) / 21.4 (1.40–44.8) | 1.22 | 34.6 (14.6–61.7) / 38.1 (6.57–86.0) | 0.91 |

| | | | | | |
|---|---|---|---|---|---|
| Gas Stoves | | | | | |
| Present | 5 (17.9) | 26.0 (19.8–31.0) / 21.1 (9.79–42.0) | 1.23 | 40.8 (14.8–61.7) / 41.2 (7.11–86.0) | 0.99 |
| Absent | 23 (82.1) | 25.6 (17.1–34.0) / 19.7 (1.40–46.8) | 1.30 | 33.4 (13.7–55.0) / 37.5 (6.57–88.5) | 0.89 |
| Stove Hood Types | | | | | |
| Exhaust | 8 (28.6) | 23.9 (17.1–29.5) / 17.6 (1.56–41.1) | 1.36 | 34.0 (15.3–52.1) / 41.0 (9.07–84.4) | 0.83 |
| Recirculating | 13 (46.4) | 25.7 (18.9–34.0) / 20.4 (1.4–46.75) | 1.26 | 34.4 (13.7–61.7) / 35.3 (6.62–86.0) | 0.97 |
| Absent | 7 (25.0) | 27.1 (19.7–32.2) / 21.5 (9.79–44.7) | 1.26 | 32.6 (14.6–59.9) / 39.8 (6.57–88.5) | 0.82 |

†BG = Built Green; EER = Energy Efficiency Retrofitted; Non-EER = Non-Energy Efficiency Retrofitted (conventional homes as a control group).
I/O ratios across all categories were statistically significant at $\alpha = 0.05$ (K-W test).

**Table S2.** Concentration comparisons between indoor and outdoor pollutants before and after data filtration.

| | Concentrations Median (Range) | | |
|---|---|---|---|
| | Raw data | Filtered data | $p_2$-value [3] |
| $PN_{0.5\text{-}2.5}$ [#/cm$^3$] | | | |
| Indoor | 1.40 (0–203) | 1.17 (0.0494–199) | <0.000 |
| Outdoor | 1.66 (0.0850–79.1) | 1.69 (0.0850–79.1) | <0.000 |
| $r_s$ [1] | 0.311 | 0.374 | |
| $p_1$-value [2] | <0.000 | <0.000 | |
| BC [ng/m$^3$] | | | |
| Indoor | 532 (82.2–15900) | 512 (82.2–5081) | <0.000 |
| Outdoor | 768 (54.5–39100) | 766 (54.5–39100) | 0.629 |
| $r_s$ | 0.507 | 0.525 | |
| $p_1$-value | <0.000 | <0.000 | |
| CO [ppm] | | | |
| Indoor | 0.485 (0–28.2) | 0.450 (0–10.5) | <0.000 |
| Outdoor | 0.150 (0–14.9) | 0.144 (0–14.0) | 0.000467 |
| $r_s$ | 0.434 | 0.252 | |
| $p_1$-value | <0.000 | <0.000 | |

[1] Spearman's rank correlation coefficient between indoor and outdoor medians.
[2] p-values for K-W test between indoor and outdoor medians.
[3] p-values for Wilcoxon Mann–Whitney (U) test between raw and filtered dataset medians (K-W test could not be performed due to different lengths of datasets).
** High value signifying outlier.

**Table 3.** Pollutant concentrations indoors and outdoors from raw datasets with the corresponding p-values from K-W test on I/O ratios between categories.

| | Concentrations: Indoor Median (Indoor Range) / Outdoor Median (Outdoor Range) | | | | | | | |
|---|---|---|---|---|---|---|---|---|
| | $PN_{0.5\text{-}2.5}$ [#/$cm^3$] | n/ (I/O†) | BC [ng/$m^3$] | n/ (I/O) | CO [ppm] | n/ (I/O) | $NO_2$ [ppb]* | n/ (I/O) |
| Location | | | | | | | | |
| Aurora | 0.992 (0.237–29.4)/ 2.47 (0.275–58.1) | 4/ **0.40** | 387 (109–3490)/ 739 (54.5–39100) | 4/ **0.52** | 0.823 (0.04–2.95)/ 0.169 (0–1.75) | 4/ **4.87** | 8.31 (6.39–13.6)/ 7.65 (4.48–9.24) | 4/ 1.08 |
| Boulder/ Longmont | 1.30 (0.0989–201)/ 1.16 (0.0848–78.7) | 7/ **1.12** | 446 (82.3–15900)/ 652 (122–11000) | 4/ **0.68** | 0.307 (0–10.4)/ 0 (0–14.0) | 9/ **-** | 5.76 (3.58–6.73)/ 5.52 (5.21–7.19) | 4/ 1.04 |
| West Denver | 1.38 (0.0494–199)/ 1.75 (0.159–79.1) | 11/ **0.79** | 672 (119–8200)/ 791 (57.9–16300) | 8/ **0.85** | 0.540 (0–28.2)/ 0.239 (0–14.9) | 11/ **2.26** | 11.9 (8.00–26.8)/ 9.89 (6.55–11.6) | 8/ 1.20 |
| Central/ North Denver | 2.10 (0–203)/ 2.38 (0.275–35.6) | 4/ **0.88** | 640 (83.3–14300)/ 957 (128–17700) | 3/ **0.67** | 0.724 (0.06–12.9)/ 0.206 (0–2.80) | 3/ **3.51** | 9.30 (8.62–13.4)/ 9.55 (7.78–20.6) | 3/ 0.97 |
| p-values: | | <0.01 | | <0.01 | | <0.01 | | 0.14 |
| Building Type | | | | | | | | |
| BG [a] | 1.41 (1.41)/ 1.03 (0.0847–78.7) | 5/ **1.37** | 480 (98.3–15900)/ 590 (176–3760) | 1/ **0.81** | 0.239 (0–4.46)/ 0 (0–5.54) | 5/ **-** | 6.73 (6.73–6.73)/ 5.21 (5.21–5.21) | 1/ 1.29 |
| EER [b] | 0.982 (0.494–199)/ 1.54 (0.159–79.1) | 11/ **0.64** | 383 (82.3–8210)/ 629 (54.5–39100) | 10/ **0.61** | 0.554 (0–28.2)/ 0.209 (0–14.0) | 13/ **2.65** | 8.03 (3.58–26.8)/ 7.37 (4.48–10.9) | 10/ 1.09 |
| Non-EER[c] | 2.57 (0–203)/ 3.23 (0.275–59.2) | 10/ **0.79** | 808 (83.3–14300)/ 961 (128–21500) | 8/ **0.84** | 0.707 (0–12.9)/ 0.217 (0–14.9) | 9/ **3.25** | 12.6 (6.48–25.5)/ 9.89 (6.07–20.6) | 8/ 1.27 |
| p-values: | | <0.01 | | <0.01 | | <0.01 | | 0.64 |
| Road Proximity | | | | | | | | |
| <100 m | 2.00 (0–203)/ 2.40 (0.131–78.7) | 15/ **0.83** | 664 (83.3–15900)/ 872 (54.5–39100) | 12/ **0.76** | 0.584 (0–12.8)/ 0.134 (0–14.9) | 15/ **4.35** | 11.8 (6.53–26.8)/ 9.59 (5.21–20.6) | 12/ 1.23 |
| 100–200 m | 0.801 (0.0494–45.0)/ 1.68 (0.159–79.1) | 5/ **0.48** | 390 (82.3–6360)/ 612 (97.0–11000) | 7/ **0.64** | 0.646 (0–4.91)/ 0.244 (0–7.88) | 7/ **2.65** | 6.48 (3.58–8.62)/ 6.55 (4.48–7.78) | 7/ 0.99 |
| >200 m | 1.17 (0.0989–199)/ 1.21 (0.0848–66.7) | 6/ **0.97** | - / - | 0 | 0.232 (0–28.2)/ 0.006 (0–14.0) | 5/ **38.67** | – / – | 0 |
| p-values: | | <0.01 | | <0.01 | | <0.01 | | 0.24 |
| Wildfire Plume Density | | | | | | | | |
| None | 0.897 (0.494–160)/ 1.10 (0.0848–79.1) | 7/ **0.81** | 424 (82.2–6360)/ 589 (98.3–6900) | 5/ **0.72** | 0.378 (0–3.23)/ 0.0755 (0–7.87) | 9/ **5.01** | 7.19 (3.58–10.1)/ 6.06 (4.48–9.24) | 6/ 1.18 |
| Low | 1.34 (0–203)/ 1.67 (0.229–66.7) | 7/ **0.80** | 568 (144–4700)/ 745 (97.0–8280) | 3/ **0.76** | 0.554 (0–12.8)/ 0.140 (0–14.9) | 7/ **3.96** | 9.87 (6.53–25.5)/ 9.58 (9.23–11.6) | 5/ 1.03 |
| Medium | 2.54 (0.251–199)/ 2.39 (0.265–59.2) | 9/ **1.06** | 644 (83.3v12100)/ 859 (54.5–39100) | 8/ **0.75** | 0.618 (0–28.2)/ 0.229 (0–1.90) | 8/ **2.70** | 10.2 (6.48–26.8)/ 8.69 (5.21–11.1) | 6/ 1.17 |
| High | 5.32 (1.36–201)/ 6.67 (1.01–58.1) | 3/ **0.80** | 540 (98.3–15900)/ 946 (176–21500) | 3/ **0.57** | 0.88 (0.26–4.46)/ 0.213 (0–2.80) | 3/ **4.13** | 13.5 (13.4–13.6)/ 13.3 (6.07–20.6) | 2/ 1.01 |
| p-values: | | <0.01 | | <0.01 | | <0.01 | | 0.70 |
| Mechanical Ventilation | | | | | | | | |

| | | | | | | | | |
|---|---|---|---|---|---|---|---|---|
| Present | 1.30 (0.198–201)/<br>1.30 (0.131–78.7) | 4/<br>**1.00** | 480 (98.3–15900)/<br>623 (98.3–6290) | 3/<br>**0.77** | 0.333 (0–4.46)/<br>0.0258 (0–7.88) | 5/<br>**12.8** | 6.73 (3.58–8.06)/<br>5.56 (5.21–6.95) | 3/<br>1.21 |
| Absent | 1.43 (0–203)/<br>1.75 (0.0847–79.1) | 22/<br>**0.82** | 557 (82.2–14300)/<br>793 (54.5–39100) | 16/<br>**0.70** | 0.539 (0–28.2)/<br>0.173 (0–14.9) | 22/<br>**3.11** | 9.99 (5.04–26.8)/<br>9.40 (4.48–20.6) | 16/<br>1.06 |
| p-values: | | <0.01 | | <0.01 | | <0.01 | | 0.65 |
| Hours Windows Open | | | | | | | | |
| 0 | 1.19 (0.314–36.8)/<br>1.57 (0.131–54.0) | 3/<br>**0.76** | 250 (98.9–6360)/<br>740 (54.4–39100) | 2/<br>**0.34** | 0.466 (0–3.23)/<br>0.0742 (0–7.88) | 3/<br>**6.28** | 5.06 (3.58–6.53)/<br>7.40 (5.56–9.23) | 2/<br>**0.68** |
| 1–6 | 0.611 (0.05–20.9)/<br>1.50 (0.159–79.1) | 4/<br>**0.41** | 316 (82.2–2120)/<br>540 (97.0–6950) | 4/<br>**0.58** | 0.713 (0–4.91)/<br>0.252 (0–3.01) | 4/<br>**2.83** | 7.20 (5.04–8.62)/<br>6.01 (4.48–7.78) | 4/<br>**1.20** |
| 7–12 | 1.24 (0.0989–160)/<br>1.41 (0.0848–78.7) | 5/<br>**0.88** | 406 (109–3000)/<br>800 (119–21500) | 2/<br>**0.51** | 0.322 (0–10.5)/<br>0 (0–14.0) | 5/<br>– | 20.2 (13.6–26.8)/<br>8.48 (6.07–10.9) | 2/<br>**2.38** |
| > 12 | 1.80 (0–203)/<br>1.82 (0.120–59.2) | 14/<br>**0.99** | 761 (83.3–15900)/<br>860 (58.0–17800) | 11/<br>**0.88** | 0.477 (0–28.2)/<br>0.179 (0–14.9) | 15/<br>**2.66** | 10.1 (6.48–25.5)/<br>9.58 (5.21–20.6) | 11/<br>**1.05** |
| p-values: | | <0.01 | | <0.01 | | <0.01 | | 0.035 |
| Gas Stove | | | | | | | | |
| Present | 1.65 (0–203)/<br>1.97 (0.275–59.2) | 5/<br>**0.84** | 597 (109–4400)/<br>858 (119–21500) | 3/<br>**0.70** | 0.736 (0–12.9)/<br>0.142 (0–1.23) | 4/<br>**5.18** | 25.5 (13.6–26.8)/<br>10.9 (6.07–11.6) | 3/<br>**2.34** |
| Absent | 1.33 (0.0494–201)/<br>1.58 (0.0848–79.1) | 21/<br>**0.84** | 522 (82.2–15900)/<br>748 (54.5–39100) | 16/<br>**0.70** | 0.435 (0–28.2)/<br>0.151 (0–14.9) | 23/<br>**2.88** | 8.34 (3.58–13.8)/<br>8.51 (4.48–20.6) | 16/<br>**0.98** |
| p-values: | | <0.01 | | <0.01 | | <0.01 | | 0.007 |
| Stove Hood | | | | | | | | |
| Exhaust | 1.18 (0.0494–201)/<br>2.40 (0.131–79.1) | 8/<br>**0.49** | 499 (98.3–15900)/<br>827 (54.4–39100) | 5/<br>**0.60** | 0.478 (0–10.4)/<br>0.160 (0–14.0) | 8/<br>**2.99** | 6.73 (6.48–13.6)/<br>7.19 (5.21–9.60) | 5/<br>0.94 |
| Recirculating | 1.31 (0.0989–160)/<br>1.27 (0.0848–78.7) | 12/<br>**1.03** | 495 (82.2–12100)/<br>695 (97.0–16200) | 10/<br>**0.71** | 0.427 (0–7.33)/<br>0.0628 (0–14.9) | 12/<br>**6.80** | 8.96 (5.04–26.8)/<br>8.51 (4.48–11.6) | 10/<br>1.05 |
| Absent | 1.85 (0–203)/<br>1.75 (0.265–53.1) | 6/<br>**1.06** | 713 (98.9–14300)/<br>904 (58.0–17800) | 4/<br>**0.79** | 0.616 (0–28.2)/<br>0.231 (0–7.88) | 7/<br>**2.67** | 10.9 (3.58–13.4)/<br>9.88 (5.56–20.6) | 4/<br>1.10 |
| p-values: | | <0.01 | | <0.01 | | <0.01 | | 0.18 |

[*] $NO_2$ concentrations were TWA measurements, all other pollutants were time resolved (one-minute resolution).
[a] BG = Built Green; [b] EER = Energy Efficiency Retrofitted; [c] Non-EER = Non-Energy Efficiency Retrofitted (conventional homes as a control group).
[†]I/O ratios that showed statistical significance (K-W test) in the mean differences ($p<0.05$) are shown in bold.

**Table 4.** Pollutant concentrations indoors and outdoors from filtered datasets with the corresponding p-values from K-W test on I/O ratios between categories.

| | Concentrations: | | Indoor Median (Indoor Range) / Outdoor Median (Outdoor Range) | | | | | |
|---|---|---|---|---|---|---|---|---|
| | $PN_{0.5-2.5}$ [#/cm[3]] | n/<br>**(I/O)** | BC<br>[ng/m[3]] | n/<br>**(I/O)** | CO<br>[ppm] | n/<br>**(I/O)** | $NO_2$<br>[ppb][*] | n/<br>**(I/O)** |
| Location | | | | | | | | |
| Aurora | 2.54 (0.680–26.2)/<br>6.13 (0.780–165) | 4/<br>**0.41** | 386 (109–3490)/<br>739 (54.5–39100) | 4/<br>**0.52** | 0.790 (0–2.95)/<br>0.185 (0–1.75) | 4/<br>**4.27** | 6.53 (6.39–10.1)/<br>9.23 (4.48–9.24) | 3/<br>0.71 |
| Boulder/<br>Longmont | 2.88 (0.280–44.8)/<br>3.42 (0.240–111) | 7/<br>**0.84** | 409 (82.3–4460)/<br>657 (122–11000) | 4/<br>**0.62** | 0.458 (0–10.5)/<br>0.156 (0–14.0) | 9/<br>**2.94** | 5.76 (3.58–6.73)/<br>9.59 (5.21–7.19) | 4/<br>0.60 |
| West Denver | 3.35 (0.140–563)/<br>5.06 (0.450–224) | 11/<br>**0.66** | 664 (119–4700)/<br>787 (57.9–16300) | 8/<br>**0.84** | 0.537 (0–5.3)/<br>0.274 (0–3.45) | 11/<br>**1.96** | 10.8 (8.00–13.8)/<br>9.89 (6.55–11.1) | 6/<br>1.1 |
| Central/<br>North Denver | 5.20 (0.790–34.8)/<br>7.14 (0.780–101) | 4/<br>**0.73** | 599 (83.3–5080)/<br>943 (128–17800) | 3/<br>**0.64** | 0.73 (0–2.18)/<br>0.279 (0–2.80) | 3/<br>**2.62** | 9.30 (8.62–13.4)/<br>9.55 (7.78–20.6) | 3/<br>0.97 |
| p-values: | | <0.01 | | <0.01 | | <0.01 | | 0.33 |
| Building Type | | | | | | | | |
| BG [a] | 2.99 (0.280–44.8)/<br>2.92 (0.240–111) | 5/<br>**1.02** | 434 (98.3–1300)/<br>564 (176–3180) | 1/<br>**0.77** | 0.335 (0–4.46)/<br>0.085 (0–4.10) | 5/<br>**3.94** | 6.73 (6.73–6.73)/<br>5.21 (5.21–5.21) | 1/<br>1.29 |
| EER [b] | 2.56 (0.140–563)/<br>4.49 (0.450–224) | 11/<br>**0.57** | 381 (82.3–3490)/<br>633 (54.5–39100) | 10/<br>**0.60** | 0.573 (0–10.5)/<br>0.242 (0–14.0) | 13/<br>**2.37** | 8.00 (3.58–11.9)/<br>6.95 (4.48–9.58) | 9/<br>1.15 |
| Non-EER[c] | 5.96 (0.280–52.4)/<br>9.08 (0.780–168) | 10/<br>**0.66** | 792 (83.3–5080)/<br>956 (128–21500) | 8/<br>**0.83** | 0.691 (0–2.95)/<br>0.248 (3.45) | 9/<br>**2.79** | 10.8 (6.48–13.8)/<br>9.89 (7.19–20.6) | 6/<br>1.09 |
| p-values: | | <0.01 | | <0.01 | | <0.01 | | 0.34 |
| Road Proximity | | | | | | | | |
| <100 m | 1.540 (0.198–15.8)/<br>2.57 (0.194–29.2) | 15/<br>**0.60** | 642 (83.3–5080)/<br>870 (54.5–39100) | 12/<br>**0.74** | 0.524 (0–4.46)/<br>0.129 (0–4.10) | 15/<br>**4.06** | 10.1 (6.53–13.8)/<br>9.58 (5.21–20.6) | 9/<br>**1.05** |
| 100–200 m | 0.685 (0.050–7.97)/<br>1.71 (0.159–79.1) | 5/<br>**0.40** | 388 (82.3–4460)/<br>614 (97.0–11000) | 7/<br>**0.63** | 0.621 (0–2.75)/<br>0.251 (0–3.01) | 7/<br>**2.47** | 6.48 (3.58–8.62)/<br>6.55 (4.48–7.78) | 7/<br>**0.99** |
| >200 m | 1.09 (0.10–198)/<br>1.27 (0.085–22.4) | 6/<br>**0.85** | - | 0/<br>- | 0.222 (0–10.5)/<br>0 (0–14.0) | 5/<br>**-** | - | 0/<br>- |
| p-values: | | <0.01 | | | | <0.01 | | |
| Wildfire Plume Density | | | | | | | | |
| None | 2.15 (0.140–44.8)/<br>3.36 (0.240–224) | 7/<br>**0.64** | 422 (82.3–3490)/<br>590 (98.3–6900) | 5/<br>**0.72** | 0.526 (0–3.23)/<br>0.224 (0–4.10) | 9/<br>**2.35** | 6.39 (3.58–10.1)/<br>5.56 (4.48–9.24) | 5/<br>1.14 |
| Low | 3.25 (0.280–52.4)/<br>4.81 (0.650–58.3) | 7/<br>**0.68** | 568 (144–4700)/<br>745 (97.0–8280) | 3/<br>**0.80** | 0.605 (0–10.5) /<br>0.206 (0–14.0) | 7/<br>**2.94** | 11.8 (8.00–13.8)/<br>9.60 (6.55–11.1) | 3/<br>1.22 |
| Medium | 6.42 (0.710–563)/<br>7.49 (0.750–168) | 9/<br>**0.86** | 632 (83.3–5080)/<br>857 (54.5–39100) | 8/<br>**0.74** | 0.528 (0–5.30)/<br>0.253 (0–1.90) | 8/<br>**2.09** | 8.96 (6.48–11.9)/<br>9.39 (7.19–10.2) | 6/<br>0.96 |
| High | 9.85 (4.08–30.4)/<br>24.9 (8.44–165) | 3/<br>**0.40** | 495 (98.3–4320)/<br>948 (176–21500) | 3/<br>**0.52** | 0.88 (0.26–4.46)/<br>0.278 (0–2.80) | 3/<br>**3.17** | 10.1 (6.73–13.4)/<br>12.9 (5.21–20.6) | 2/<br>0.78 |
| p-values: | | <0.01 | | <0.01 | | <0.01 | | 0.46 |
| Mechanical Ventilation | | | | | | | | |
| Present | 2.95 (0.560–44.8)/ | 4/ | 448 (98.3–2290)/ | 3/ | 0.442 (0–4.46)/ | 5/ | 6.73 (3.58–8.06)/ | 3/ |

| | | | | | | | | |
|---|---|---|---|---|---|---|---|---|
| | 3.71 (0.550–111) | **0.80** | 622 (98.3–6290) | **0.72** | 0.180 (0–4.10) | **2.46** | 5.56 (5.21–6.95) | 1.21 |
| Absent | 3.43 (0.140–563)/ | 22/ | 541 (82.3–5080)/ | 16/ | 0.602 (0–10.5)/ | 22/ | 9.30 (5.04–13.8)/ | 13/ |
| | 5.03 (0.240–224) | **0.68** | 787 (54.5–39100) | **0.69** | 0.237 (0–1.40) | **2.54** | 9.24 (4.48–20.6) | 1.01 |
| p-values: | | <0.01 | | <0.01 | | <0.01 | | 0.94 |
| Hours Windows Open | | | | | | | | |
| 0 | 1.13 (0.314–6.71)/ | 3/ | 219 (98.9–924)/ | 2/ | 0.385 (0–3.23)/ | 3/ | 5.05 (3.58–6.53)/ | 2/ |
| | 2.44 (0.194–54.0) | **0.46** | 784 (54.5–39100) | **0.28** | 0.0582 (0–2.89) | **6.61** | 7.39 (5.56–9.23) | 0.68 |
| 1–6 | 0.544 (0–4.31)/ | 4/ | 317 (82.2–2120)/ | 4/ | 0.692 (0–2.75)/ | 4/ | 7.19 (5.04–8.62)/ | 4/ |
| | 1.49 (0.160–79.1) | **0.36** | 540 (97–6950) | **0.59** | 0.258 (0–3.01) | **2.68** | 6.01 (4.48–7.78) | 1.20 |
| 7–12 | 0.932 (0.10–15.8)/ | 5/ | 406 (109–3000)/ | 2/ | 0.301 (0–10.4)/ | 5/ | - | 0 |
| | 1.48 (0.085–58.1) | **0.63** | 800 (119–21500) | **0.51** | 0 (0–14.0) | - | | |
| >12 | 1.50 (0.10–199)/ | 14/ | 739 (83.3–5100) | 11/ | 0.448 (0–5.30)/ | 15/ | 9.98 (6.48–13.8)/ | 10/ |
| | 1.78 (0.120–59.2) | **0.84** | 857 (58–17700) | **0.86** | 0.175 (0–3.45) | **2.56** | 9.56 (5.21–9.23) | 1.05 |
| p-values: | | <0.01 | | <0.01 | | <0.01 | | 0.11 |
| Gas Stove | | | | | | | | |
| Present | 1.47 (0.100–18.5)/ | 5/ | 597 (109–4400)/ | 3/ | 0.672 (0.08–2.95)/ | 4/ | - | - |
| | 2.02 (0.275–59.2) | **0.73** | 858 (119–21500) | **0.70** | 0.147 (0–1.13) | **4.57** | | |
| Absent | 1.11 (0.050–199)/ | 21/ | 503 (82.3–5080)/ | 16/ | 0.420 (0–10.4)/ | 23/ | 8.34 (3.58–13.8)/ | 16/ |
| | 1.60 (0.085–79.1) | **0.69** | 745 (54.5–39100) | **0.67** | 0.144 (0–14.0) | **2.92** | 8.51 (4.48–20.6) | - |
| p-values: | | <0.01 | | <0.01 | | <0.01 | | |
| Stove Hood Type | | | | | | | | |
| Exhaust | 0.978 (0.05–9.24)/ | 8/ | 479 (98.3–4460)/ | 5/ | 0.473 (0–10.4)/ | 8/ | 6.63 (6.48–11.8)/ | 4/ |
| | 2.39 (0.160–79.1) | **0.41** | 829 (54.4–39100) | **0.58** | 0.159 (0–14.0) | **2.97** | 8.21 (5.21–9.60) | 0.81 |
| Recirculating | 1.04 (0.10–15.8)/ | 12/ | 490 (82.3–5080)/ | 10/ | 0.389 (0–2.75)/ | 12/ | 8.34 (5.04–13.8)/ | 8/ |
| | 1.24 (0.085–59.2) | **0.81** | 693 (97.0–16200) | **0.71** | 0.050 (0–4.10) | **7.78** | 7.37 (4.48–11.1) | 1.13 |
| Absent | 1.62 (0.10–199)/ | 6/ | 685 (99.0–4320)/ | 4/ | 0.592 (0–5.30)/ | 7/ | 10.9 (3.58–13.4)/ | 4/ |
| | 1.76 (0.264–53.1) | **0.92** | 908 (57.9–17700) | **0.75** | 0.258 (0–2.80) | **2.29** | 9.88 (5.56–20.6) | 1.10 |
| p-values: | | <0.01 | | <0.01 | | <0.01 | | 0.30 |

[*] $NO_2$ concentrations were TWA measurements, all other pollutants were time resolved (one-minute resolution). Data from homes with gas stoves were not included in the filtered dataset.

[a] BG = Built Green; [b] EER = Energy Efficiency Retrofitted; [c] Non-EER = Non-Energy Efficiency Retrofitted (conventional homes as a control group).

I/O ratios that showed statistical significance (K-W test) in the mean differences (p<0.05) are shown in bold.
